# Role of Alkali Cations in DNA–Thioflavin T Interaction

**DOI:** 10.1021/acs.jpcb.4c02417

**Published:** 2024-06-04

**Authors:** P. Hanczyc

**Affiliations:** Institute of Experimental Physics, Faculty of Physics, University of Warsaw, Pasteura 5, Warsaw 02-093, Poland

## Abstract

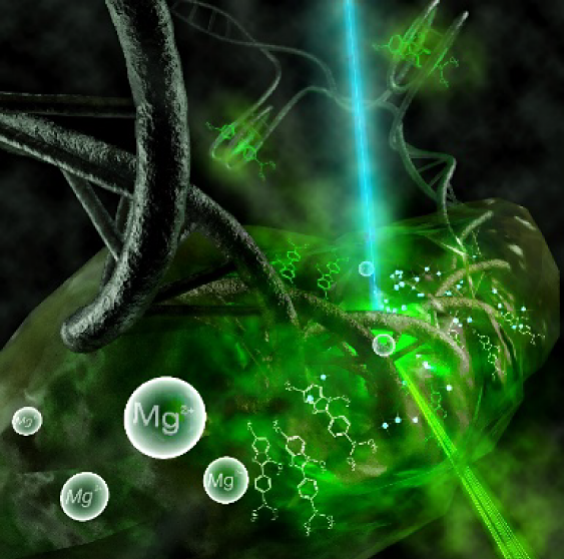

This study investigates the role of alkali cations in modulating the interaction between deoxyribonucleic acid (DNA) and Thioflavin T (ThT) in dilute and condensed phases. The emission characteristics of ThT were analyzed in the presence of double-stranded DNA and G-quadruplex structures with a focus on the effects of four cations: sodium, potassium, calcium, and magnesium. The ThT emission in double-stranded DNA was influenced by direct DNA binding and steric hindrance within the hydration shell of DNA, which was modulated by the presence of alkali cations. Lasing spectroscopy experiments further highlighted ThT sensitivity to the spatial arrangement of water molecules in the DNA hydration shell. Lasing was exclusively observed in the presence of Mg^2+^ in the G-quadruplex structure, suggesting that the parallel propeller configuration of G4 provides an optimal environment for ThT light amplification. This study highlights the critical role of cations in DNA–dye interactions and reaffirms the significance of ThT in biophysical studies.

## Introduction

The structural and functional dynamics of deoxyribonucleic acid (DNA) are significantly modulated by the presence of metal ions.^1,2^ Alkali cations are important for DNA because the negative charges of the phosphate groups in the DNA backbone are neutralized by the cations to increase the stability of the double helix.^3^ Another structural motif where alkali cations play a central role is G-quadruplexes (G4s).^4^ G4s are responsible for tumor cell development, gene regulation, and cell division.^5,6^ Cations (K^+^ and Mg^2+^) effectively stabilize G4 structures.^7^ In addition, cations play a vital role in DNA replication^8^ and repair^9^ processes. Divalent ions can facilitate the accurate positioning of catalysts between DNA and enzymes for catalytic mechanisms.^10^ Several DNA-binding proteins require cations to bind to DNA during DNA–protein interactions.^11^ Thus, the significance of alkali cations in the structural and functional dynamics of DNA is undeniable.

**Figure 1:**
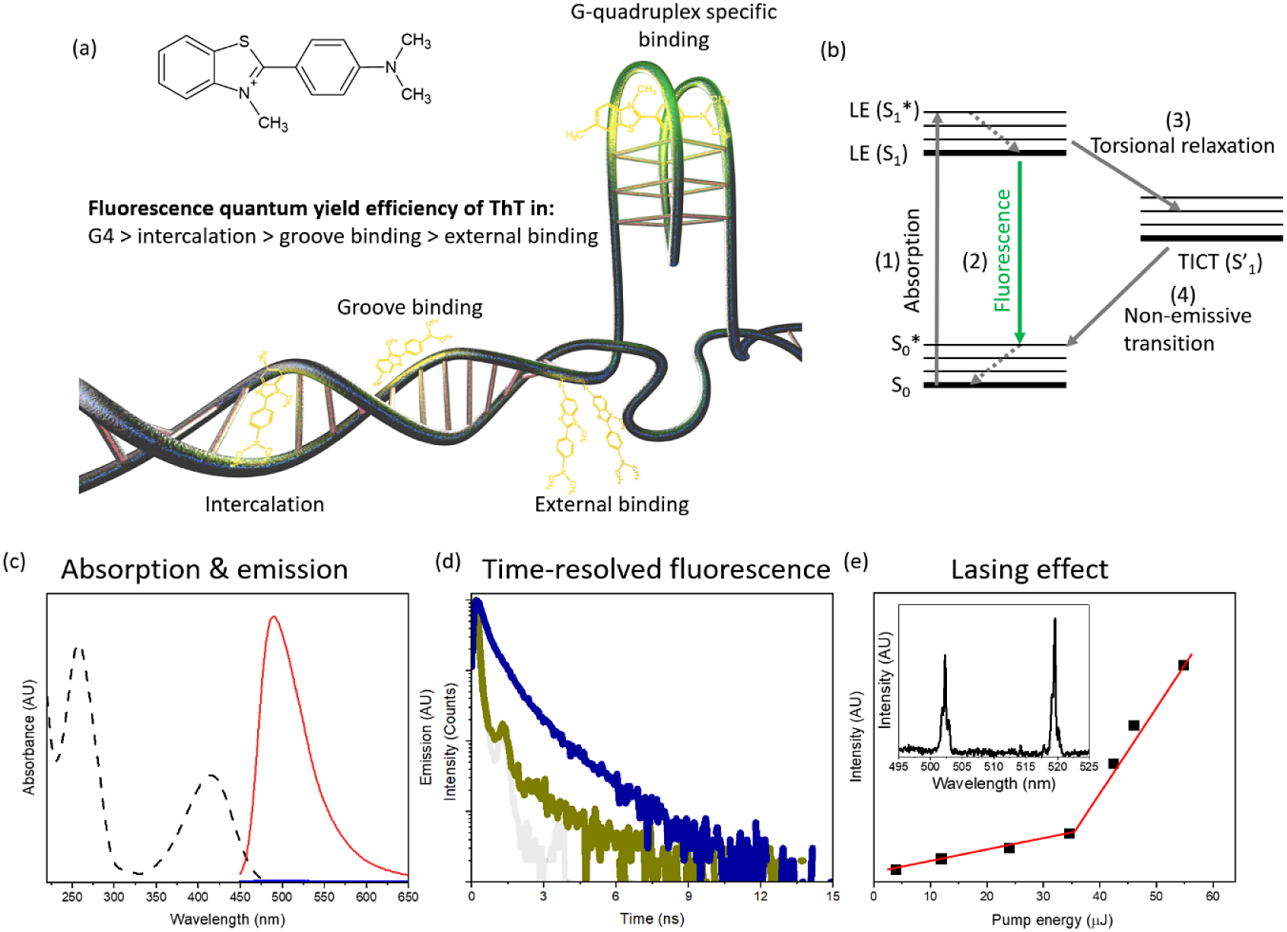
(a) Schematic illustration of the possible binding modes and interaction between ThT and DNA structures - duplex and G-quadruplex. Green color illustrates the fluorescence intensity strongly linked with the type of interaction between DNA and ThT. (b) Diagram illustrating the deactivation pathways of excitation energy of ThT. The arrows indicate (1) energy absorption, (2) fluorescence emission from the LE state, (3) torsional adjustment transitioning from the LE state to TICT state, and (4) a nonemissive return from the TICT state to the ground state. (c) Absorption (black dashed) and emission of pristine ThT (blue solid) and emission in the presence of DNA duplex (red solid). (d) Fluorescence decays of pristine ThT (dark yellow), duplex DNA–ThT (dark blue), and IRF (gray). (e) Lasing threshold of duplex DNA–ThT. The insets represent the lasing spectrum measured in the Fabry–Perot cavity at a pump energy that is distinct from the lasing threshold. Excitation is set at 430 nm.

The DNA structure and dynamics and the role of alkali cations can be studied by the addition of external chemical probes.^12−14^ DNA–dye interactions represent a fundamental aspect of biophysical research.^15^ The interaction between DNA and specific fluorophores modulates photophysical properties, such as altered fluorescence emission or absorption characteristics.^16^ Two typical modes of binding are prevalent: (i) intercalation where the dye molecule is inserted between DNA base pairs and (ii) groove binding where the dye associates with the minor or major DNA groove.^17^ The specificity and affinity of this interaction are dependent on the molecular structure of the dye, nucleotide sequence, tertiary structure of DNA, and ionic strength and composition of the surrounding milieu. Figure 1a shows the possible binding modes and interactions between the dye molecules and DNA.

G4 is an intriguing DNA structure that interacts with dyes.^18^ Several fluorophores preferentially bind to G4 than double-stranded DNA (dsDNA)^19−22^ and manifest enhanced fluorescence after binding, enabling the detection and study of G4 in various biological, chemical, and environmental contexts.^23−25^ The interaction between dyes and G4 structures provides insight into their structure, topology, dynamics, and microenvironment.^26,27^ Thioflavin T (ThT) is extensively used for probing G4 because it remarkably enhances fluorescence in the presence of G4.^28^ ThT belongs to the group of molecular rotors that show strong emission when ring rotation is inhibited on adopting a specific chemical configuration.^29^ The photophysical mechanism of ThT fluorescence is related to an ultrafast torsional motion that causes nonradiative deactivation owing to twisted intramolecular charge transfer (TICT).^30^ Fluorescence appears from the locally excited (LE) state when TICT is blocked (Figure 1b). Negligible fluorescence in water owing to TICT increases when ThT interacts with DNA (Figure 1c).^31^ Although ThT–H_2_O in water has an ultrafast relaxation time as indicated via time-resolved fluorescence measurements, DNA–ThT has a lifetime of nanoseconds (Figure 1d).^32^ The DNA environment and the inhibition of ThT ring rotation are perfect conditions for the classical four-level energy system, which enables population inversion for lasing from the LE state.

The Fabry–Perot cavity lasing uses a pair of mirrors functioning as photonic resonators for light amplification within the cavity.^33^ A distinct lasing peak emerges when the energy within the cavity surpasses the threshold energy required for population inversion.^34^ The lasing phenomenon is evident, as the excitation energy is incrementally elevated, as shown in Figure 1e. The pump energy at which this transition occurs is termed light amplification or the lasing threshold. Beyond the threshold, the emission spectrum narrows significantly, resulting in a full width at half-maximum of the lasing spectrum spanning for a few nanometers (inset in Figure 1e) accompanied by a substantial rise in light intensity.

The mirror cavity provides strong optical feedback that can be used to study DNA–dye interactions, where bound ThT is the gain medium.^35^ The traditional spectroscopic techniques of steady-state fluorescence (SSF), time-resolved fluorescence (TRF), and linear dichroism (LD) primarily focus on the examination of DNA in diluted solutions. Lasing spectroscopy utilizes the Fabry–Perot cavity with a different approach to examine the DNA structure and dynamics in the condensed phase.

ThT binds with dsDNA using multiple modes, such as intercalation, groove binding, and external binding, as evidenced by various studies.^36−38^ The balance between these binding modes plays a pivotal role in determining the quantum yield efficiency.^38^ Intercalation restricts the rotation of the dye rings and increases the fluorescence. Alternately, external binding causes fluorescence quenching when dye molecules are in the proximity (Å) of intercalated ThT, resulting in homofluorescence resonance energy transfer (homoFRET). A similar quenching effect can arise owing to the dimerization of ThT within major grooves.^39^

Four guanine units combine via Hoogsteen hydrogen bonds in a G4 structure. Each guanine unit acts as a donor and acceptor for two hydrogen bonds. Computational simulations revealed that ThT can accommodate inside the G4 structure or groove.^40^

The multiple binding property of ThT to DNA is used for sensing in chemical or biological sensors.^41^ Feng et al.^42^ used a ThT–G4 complex for sensing radioactive strontium ions. Pramanik et al.^43^ differentiated single nucleotide mismatch via metal ion-mediated base pairing in ThT-stained DNA. However, there is a gap in understanding the influence of common cations on the DNA–ThT complex.

In previous work by Hanczyc et al.,^38^ it was found that competitive interaction modes of ThT within DNA are critically affecting dye fluorescence quantum yield. The article discussed how these modes impact ThT’s quantum yield in DNA structures, highlighting that external binding leads to fluorescence quenching due to energy transfer between intercalated and externally bound molecules. This complex interplay was crucial in generating population inversion at different pumping energy thresholds offering structural insights on DNA–ThT complexation from amplified emission effects in the solid-state.

This work is a progression from the foundational understanding of ThT–DNA interaction modes to their complex modulation by specific cations in two solution states: a diluted solution and a condensed highly concentrated solution. Results and discussion on interaction modes is expanded to include the modulatory effects of alkali cations (Na^+^, K^+^, Ca^2+^, and Mg^2+^) on dsDNA–ThT and G4-ThT interactions. The article addresses how specific cations influence ThT’s photophysical properties, particularly in terms of fluorescence emission and lasing behavior. The findings underline the complexity of ThT’s interaction with DNA, dependent not just on the structural binding modes but also significantly influenced by the ionic environment. Ions compete with ThT for the binding sites, leading to dye suspension in the DNA hydration shell. The change on interaction mode affects fluorescence quantum yield and thus lasing efficiency, opening for a more detailed analysis of structural arrangement of dye molecules in the DNA hydration shell.

Overall, the emission properties of ThT were examined in the presence of dsDNA and G4 with four alkali cations (Na^+^, K^+^, Ca^2+^, and Mg^2+^) in this study via LD, SSF, TRF, and lasing to probe nucleic acid structures in dilute and condensed phases.

## Materials and Methods

### Thioflavin T

Ultrapure grade Thioflavin T (ThT) was procured from AATBioquest. For standard fluorescence experiments, a stock solution was prepared using distilled water to achieve 3.14 mM whereas for cavity lasing experiments, the dye stock solution was 78.4 mM.

### Double-Stranded Calf Thymus DNA (type I)

It, referred to as the dsDNA duplex throughout the main text, was obtained from Sigma-Aldrich. It was solubilized in distilled water. Then appropriate salt was added (NaCl, KCl, MgCl_2_, or CaCl_2_) to achieve a final salt concentration of 20 mM. Two DNA set of concentrations were prepared: 0.78 mM and 1.56 mM for fluorescence and linear dichroism and 37 mM and 74 mM for lasing experiments. Concentrations of the dsDNA solutions were calculated based on the average mass of an individual base (330 g/mol).

### G-Quadruplex (G4 DNA)

The sequence of the G4 was: 5′-GGGG TTTT GGGG TTTT GGGG TTTT GGGG TTTT GGGG-3′. Lyophilized G4 was dissolved in 20 mM of designated salt (NaCl, KCl, MgCl_2_, or CaCl_2_). Concentrations of G4 were 0.78 mM for fluorescence and 37 mM and 74 mM for lasing experiments. To facilitate the formation of the G4 structures, the G-quadruplex sequence was heated to 95 °C for a duration of 3 min and subsequently cooled gradually to ambient temperature.

### Cavity

The mirrors used creating an optical cavity for lasing displayed nearly 100% transmission in the 400–450 nm range, while the reflectance within the 470–570 nm spectrum was approximately 95–99%, peaking between 520 and 530 nm. The sample, which was the gain medium in the cavity, was prepared by mixing DNA and ThT in volume ratio 2:1. For experimental purposes, DNA was combined with the dyes in a volume ratio of 2:1 (DNA:dye) for cavity lasing. DNA dye mixture led to a cavity thickness of around 8 μm.

### UV–Vis Spectroscopy

Absorption spectra were obtained by using a CARY-5000 spectrophotometer.

### Linear Dichroism

The Chirascan CD spectrophotometer was used to obtain linear dichroism (LD) spectra. LD denotes the absorbance difference between light that is linearly polarized in parallel (*A*_∥_) and perpendicular (*A*_⊥_) directions relative to the DNA’s orientation axis.
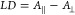


To achieve this orientation, a unit flow Couette cell was employed. The Couette cell had a total path length of 1 mm. The sample underwent shear alignment within a 0.5 mm gap between two aligned quartz cylinders inside a tailor-made outer-rotating Couette cell with a shear gradient of 3100 s^–1^.

### Fluorescence Spectroscopy

Fluorescence spectra and time-resolved decays were measured using a Horiba system QuantaMaster 8075–11 Spectrofluorometer. Samples were excited using a PowerArc instrument at wavelength 430 nm. The slits for excitation and emission were adjusted to 1 nm. Fluorescence was collected perpendicular to the excitation light direction. The solution-containing cuvette was positioned at a 90° angle. Time-resolved decays were collected in an analogous experimental configuration to steady-state experiments but the exciation source was a femtosecond laser operating at a repetition rate of 2 MHz and delivering a pulse energy of 10 μJ. Recorded fluorescence kinetics were analyzed by fitting with multiexponential decay functions convoluted with the IRF (measured by scattering the excitation light in a suspension of titanium dioxide) using the Horiba FelixGX software. The average fluorescence lifetime was calculated as the amplitude-weighted mean of the decay times of each component.

### Cavity Lasing

Lasing spectra were obtained utilizing a femtosecond laser that operates at a repetition rate of 0.5 kHz and delivers a pulse energy of 400 μJ. An optical amplifier was used to tune the wavelength to 430 nm, which was used for excitation. This adjustment was essential to match the specifications of cavity mirrors and the sample excitation spectrum. A depiction of the experimental setup for these lasing experiments is provided in the Supporting Information.

## Results and Discussion

Figure 1a,b illustrates the possible interaction between ThT and DNA, and the dye relaxation mechanism. The photophysical characterization of ThT-stained dsDNA is shown in Figure 1c–e. TRF spectrum of pristine ThT shows ultrafast relaxation below the instrumental response function (IRF), whereas ThT bonded to dsDNA increases the fluorescence lifetime with decay to nanoseconds (Figure 1d). Figure 1e indicates the lasing threshold of dsDNA–ThT sandwiched between two mirrors, creating a lasing cavity. The inset shows the spectrum above the lasing threshold. SSF, TRF, and lasing techniques were used to study the influence of Na^+^, K^+^, Ca^2+^, and Mg^2+^ on the photophysics of dsDNA–ThT and G4-ThT.

**Figure 2:**
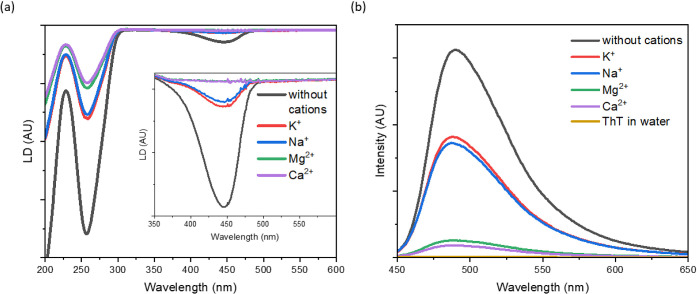
(a) LD of dsDNA–ThT oriented in shear flow without salt (black) and with K^+^ (red), Na^+^ (blue), Mg^2+^ (green), and Ca^2+^ (violet) salts. The inset shows the LD spectrum enlarged in the spectral range of the main absorption band of ThT. *C*_DNA_ = 0.78 mM, *C*_ThT_ = 4 μM, and concentration of cations was 20 mM. (b) Fluorescence spectra of dsDNA–ThT in the presence of K^+^ (red), Na^+^ (blue), Mg^2+^ (green), and Ca^2+^ (violet) *C*_DNA_ = 0.78 mM, *C*_ThT_ = 13 pM, and concentration of cations was 20 mM. DNA with salts were incubated for 2 min prior the measurements. As a reference, the graph includes the emissions of dsDNA–ThT without salt (black) and pristine ThT in water (dark yellow).

dsDNA was examined via LD (Figure 2a). The LD spectrum of the shear flow-oriented dsDNA–ThT complex provides information about the orientation of the bound dye molecules relative to the DNA helix axis. The nonzero LD values are obtained owing to intercalation and groove binding, whereas external binding and noninteracting dye molecules exhibit zero LD. The nucleobases of dsDNA and ThT of dsDNA–ThT in the absence of salt absorb at 260 and 440 nm, respectively, with a negative LD signal, indicating a perpendicular orientation of nucleobases and dye molecules concerning the DNA helix axis. Thus, dye molecules and nucleobases are in the same plane, proving DNA intercalation. The addition of monovalent Na^+^ and K^+^ reduces the LD signal of ThT at the dye absorption band substantially. The LD signal of Ca^2+^ and Mg^2+^ is not detected for ThT at the dye absorption band.

Alkali cations screen the negatively charged phosphate groups, stabilizing the DNA helix. The simultaneous addition of dye and salt molecules competes for binding sites between ions and fluorophores. ThT can bind externally to phosphate groups where ions counteract with phosphate.^38^ LD spectra confirm that the addition of salt reduces the level of intercalation of dye molecules into the DNA helix.

The LD results agree with those of SSF. The addition of salt suppresses ThT emissions (Figure 2b). Although fluorescence is detected in the presence of Mg^2+^ and Ca^2+^, no LD signal is detected. Thus, either ThT is reconfigured into the external binding mode or ThT molecules remain in proximity to the DNA helix in the hydration shell with microviscosity higher than water, which hinders the ring rotation of ThT responsible for fluorescence. Thus, cations modulate the fluorescence quantum yield efficiency of ThT complexed with dsDNA.

**Figure 3:**
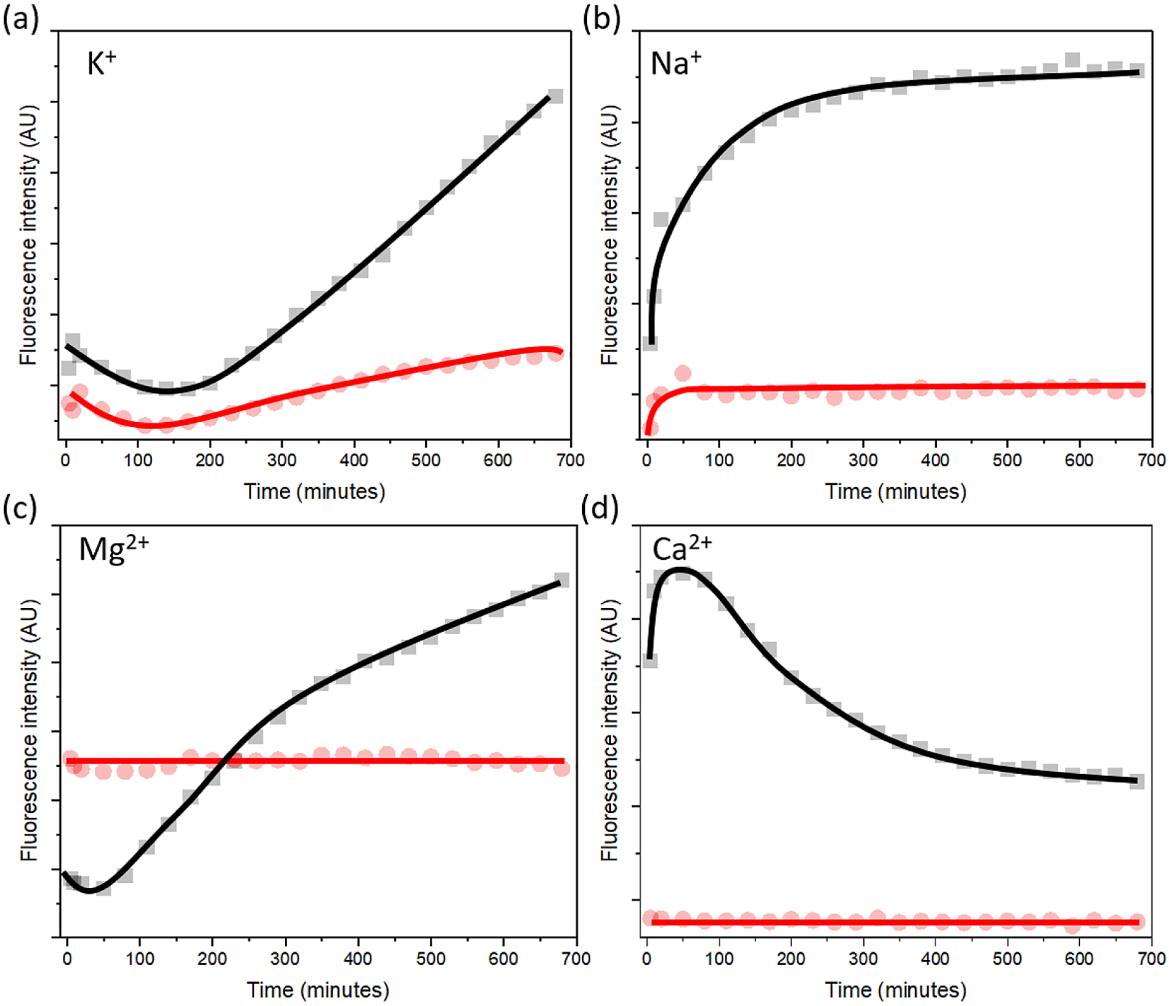
Emission kinetics of ThT doped to dsDNA with two different concentrations: 0.78 mM (**1**) (red dots), [dye:pase pairs] 1:100, and 1.56 mM (**2**) (black squares), [dye:pase pairs] 1:200 in the presence of 20 mM of (a) K^+^, (b) Na^+^, (c) Mg^2+^, and (d) Ca^2+^. DNA with salts were incubated for 2 min prior the measurements. ThT concentration is constant at 13 pM. Excitation wavelength is 430 nm.

The hypothesis of dye molecule reconfiguration between binding modes or the potential suspension of ThT in the hydration shell in the presence of cations was investigated by performing fluorescence kinetic experiments using nucleic acids of concentrations of 0.78 mM (**1**) and 1.56 mM (**2**). The ThT concentration (13 pM) was constant (Figure 3) and was kept low to avoid self-aggregation of the dye.^44^ Recalculating the concentrations for [dye:base pairs] ratios, there is one dye molecule per 100 bp in conjugate (**1**) and one dye molecule per 200 bp in conjugate (**2**), respectively. In (**1**) and (**2**), the trend of fluorescence kinetics of ThT with Na^+^ and K^+^ ions is similar. In the presence of Na^+^, emission initially increased during the first hour, and the fluorescence signal was stabilized over the next hours. In the case of K^+^, emissions initially decreased, followed by an increase in fluorescence after 3 h. At [dye:base pairs ratio] 1:200 (**2**), higher fluorescence quantum yield was observed for Na^+^ and K^+^ than for ratio 1:100 (**1**).

Emission trends of ThT with Ca^2+^ and Mg^2+^ were stable over experiment time in ThT-DNA conjugate (**1**) meaning that the system is in equilibrium from the start.

In contrast, in ThT-DNA conjugate (**2**) emission in the presence of Mg^2+^ initial decreases, followed by a substantial increase in fluorescence after 2 h. The emissions with Ca^2+^ showed an opposite kinetic trend with an initial rise of fluorescence within the first 2 h followed by a gradual decline.

Overall, ThT shows a time-dependent fluorescence in the presence of the four cations for system (**2**) but only for Na^+^ and K^+^ for system (**1**) meaning that unless there is an equilibrium at the binding sites from the start, the dye molecules tend to reorganize between binding modes due to dye-cation competition. The exact mechanism of reorganization is driven by specific cations and the number of available DNA binding sites (dye:base pairs ratio).

Emission intensity is high in samples with low dye contents relative to base pairs in the two studied ratios. The significant difference in emission intensity at low ratios can be attributed to homoFRET arising from dsDNA–ThT and molecules suspended in the hydration shell or free in the solvent. A statistical quenching of free molecules in the solvent is negligible at the picomolar concentration. Hence, dyes in the hydration shell of DNA might be exclusively involved in homoFRET.^45^

Assuming that the quantum yield of ThT is linked with the inhibition of ring rotation, the emission of dsDNA–ThT would be the highest for intercalated ThT, followed by external binding, where ThT would be less strictly confined. The confinement of unbound dye molecules in the hydration shell exhibits emission owing to microviscosity with no detectable emission in a water solvent.

Stationary methods were accompanied by the time-resolved analysis of cation effects in both the dsDNA: ThT ratios. The average lifetimes were longer in the sample with low dsDNA: ThT (**2**) ratio where less quenching occurs from unbound ThT molecules. The results agree well with the hypothesis that two mechanisms are responsible for ThT fluorescence: binding to DNA and microviscosity in the hydration shell, where unbound ThT molecules exhibit some weak fluorescence.

Similar to SSF, the time-resolved experiment revealed that ThT lifetimes are strongly influenced by alkali cations. ThT lifetimes are longer in the presence of Mg^2+^ and Ca^2+^ than those in K^+^ and Na^+^ (Table 1). Thus, it is anticipated that a long fluorescence lifetime might correspond to strong fluorescence intensity, but it is the opposite. The fluorescence intensity is the strongest in the absence of salt, whereas it is the lowest in the presence of magnesium and calcium.

**Table 1:** Lifetime of ThT-Stained dsDNA with 20 mM Salts During Excitation at 430 nm with Emissions Collected at 490 nm

		lifetimes				lifetimes		
	C_DNA_ 0.78 mM(**1**)	τ_1_ (ns)	τ_2_ (ns)	τ_avg_ (ns)	C_DNA_ 1.56 mM(**2**)	τ_1_ (ns)	τ_2_ (ns)	τ_avg_ (ns)
No salt		0.45	1.54	0.71		0.48	1.569	0.89
		±0.01	±0.01			±0.01	±0.01	
		(0.76)	(0.24)			(0.59)	(0.41)	
K^+^		0.48	1.569	0.93		0.47	1.569	0.94
		±0.01	±0.01			±0.01	±0.01	
		(0.64)	(0.36)			(0.58)	(0.42)	
Na^+^		0.38	1.59	0.76		0.49	1.91	0.98
		±0.01	±0.01			±0.01	±0.01	
		(0.65)	(0.35)			(0.69)	(0.32)	
Mg^2+^		0.50	2.14	1.17		0.60	2.43	1.43
		±0.01	±0.01			±0.01	±0.01	
		(0.59)	(0.41)			(0.59)	(0.41)	
Ca^2+^		0.47	2.12	1.22		0.57	2.38	1.44
		±0.01	±0.01			±0.01	±0.01	
		(0.52)	(0.48)			(0.55)	(0.45)	

The LD results show the complete elimination of ThT intercalation in the presence of Mg^2+^ and Ca^2+^. The lifetime is attributed to changes in the microviscosity in the hydration shell, where ThT probably resides. The microenvironment can hinder nonradiative decay processes to a certain extent so that emission would be visible, but it is less intense than the fluorescence observed in dsDNA–ThT owing to intercalation.

Divalent cations, owing to their stronger electrostatic interaction than monovalent ions, can induce significant reorganization of the surrounding water molecules. This results in the formation of a structured hydration shell, characterized by a high degree of order and potentially increased viscosity compared with bulk water. ThT molecules close to the DNA backbone (interacting through external binding) or in the hydration shell are subjected to a different microenvironment than those in bulk water or intercalated into DNA. Thus, the SSF and TRF results of the dsDNA–ThT system in the presence of cations are between the two extreme conditions of strong (DNA–ThT intercalation) and minimal (ThT in water) emissions.

The hypothesis of microviscosity in the hydration shell was further explored. ThT (26.1 mM) was dissolved in 99% and 96% ethanol and analyzed via lasing spectroscopy in cavities (a schematic illustration of the setup for lasing experiments is shown in Figure S1. This method is a definite test to examine the subtle changes in the microenvironment surrounding ThT. The sample in 99% ethanol exhibited lasing, whereas that in 96% ethanol failed to do so (results not shown). This contrast between the two samples with a difference due to 3% of the water content shows remarkable sensitivity of ThT to water molecule organization. No lasing in 96% ethanol indicates that water molecules preferentially create a shell around the dye molecules. Thus, the ultrafast nonradiative decay channel is dominant and prevents the population inversion required for laser action.

**Figure 4:**
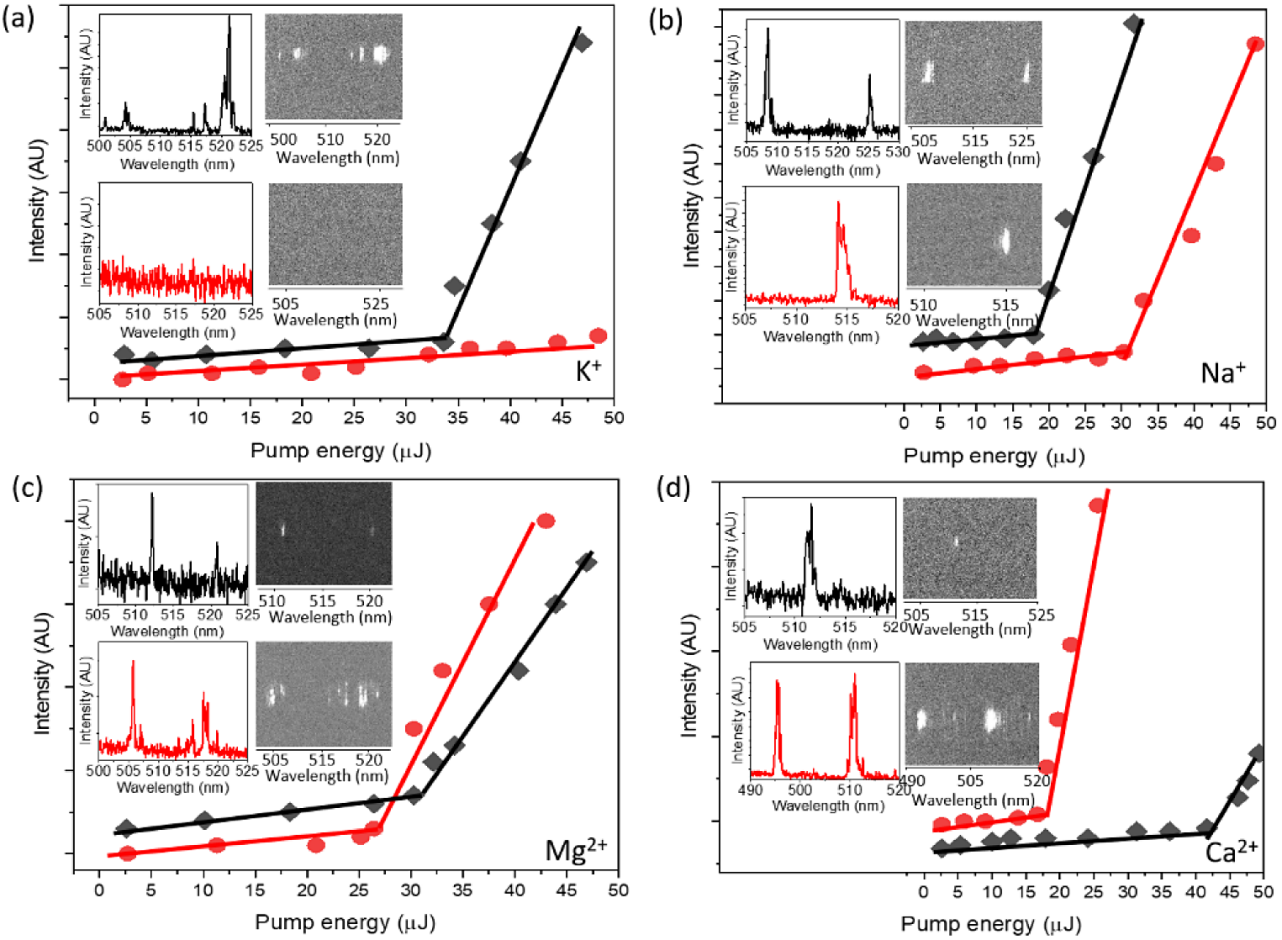
Lasing thresholds of ThT-doped dsDNA at concentrations of 22.4 mM (red dots) and 44.8 mM (black diamonds) in the presence of 20-mM (a) K^+^, (b) Na^+^, (c) Mg^2+^, and (d) Ca^2+^. ThT concentration is constant at 26.1 mM. The insets show the lasing spectra and images. Excitation wavelength is 430 nm.

The lasing experiments were next conducted in the dsDNA–ThT mixture, where the ThT concentration was 26.1 mM and the DNA concentration was 22.4 and 44.8 mM (Figure 4). DNA is a viscous gel in these concentrations, indicating a large contraction of nucleic acid. The interspace distance between DNA molecules is shorter in the condensed phase than those in the dilute phase. Thus, the condensed phase should enhance ThT emissions arising from unbound fluorophores trapped in the hydration shell. The lasing experiments with ThT in 99% and 96% ethanol show that water preferably surrounds ThT. Thus, cations that differ in the number of water molecules surrounding them should affect ThT lasing characteristics and the lasing threshold.

Lasing in the dsDNA–ThT mixture was measured in the presence of K^+^, Na^+^, Mg^2+^, and Ca^2+^. Lasing was not detected in the 22.4 mM DNA in the presence of K^+^. K^+^ attracts a large number of water molecules and traps ThT in hydration cavities with a structure similar to that of bulk water. The results of ThT lasing in 99% and 96% ethanol prove the hydration cavities. Additional evidence of the unique role of K^+^ on ThT is obtained from the dye lifetime. The lifetime measured in a dilute phase shows that fluorescence lifetimes are identical for samples with 1 dye molecule: 100 bp **(1)** and 1 dye molecule: 200 bp **(2)** ratios (Table 1). It indicates that K^+^ creates a specific microenvironment around ThT in the hydration shell, which is independent of the DNA: dye ratio and DNA concentration.

Increasing the DNA concentration to 44.8 mM restored the lasing signal of dsDNA–ThT in the presence of K^+^ ions. Probably macroscopic viscosity, an additional factor, creates favorable conditions for lasing at high concentrations, which agrees well with the results of the lasing thresholds in the presence of Na^+^. An increase in DNA concentrations and viscosity causes a significant reduction of pump energy required for obtaining lasing. The energy required to obtain lasing of 22.4 mM and 44.8 mM dsDNA is 30.3 μJ and 19.4 μJ, respectively. The LD results indicate that K^+^ and Na^+^ do not eliminate ThT from intercalation sites completely. Thus, it can be anticipated that there would be an increased availability of DNA intercalation sites at high DNA concentrations, which might mitigate some disruptive effects of cations to reduce the lasing threshold under conditions unfavorable at low DNA concentrations.

An opposite effect is observed for dsDNA–ThT in the presence of Mg^2+^ and Ca^2+^. Excess pumping energy is required to obtain the lasing of ThT fluorophores with high DNA concentrations. The lasing thresholds of ThT are 27.8 and 17.1 μJ in the presence of Mg ^2+^ and Ca ^2+^, respectively, with a DNA concentration of 22.4 mM. However, the lasing thresholds of ThT increase to 32 μJ and 44.4 μJ in the presence of Mg ^2+^ and Ca ^2+^, respectively, when the DNA concentration is increased to 44.8 mM. The lasing thresholds are presented in Table 2.

**Table 2:** Lasing Thresholds of ThT-Stained dsDNA and G4 with 20-mM Salts^a^

	lasing threshold (μJ)	
**dsDNA**	22.4 mM	44.8 mM
K^+^		33.7 ± 1.5
Na^+^	30.3	19.4 ± 4.2
Mg^2+^	27.8	32.0 ± 0.4
Ca^2+^	17.1	44.4 ± 1.3
**G4**		
K^+^		
Na^+^		
Mg^2+^		27.7 ± 0.5
Ca^2+^		

a Excitation was at 430 nm.

Mg^2+^ and Ca^2+^ thwart DNA intercalation indicated by the LD results shown in Figure 2a. Two influential factors have to be considered: macroviscosity and intricate microenvironmental changes within the hydration shell. The increased pump energy required for lasing of ThT in dsDNA–ThT with a high DNA concentration (44.8 mM) suggests that the broadening effects of macroviscosity might be secondary to the more localized changes in the microenvironment of hydration shells in the presence of divalent ions.

Lower lasing thresholds at 22.4 mM DNA suggest that at a higher dye-to-base pair ratio than in 44.8 mM, divalent cations can organize ThT molecules in the hydration shell. Such an alignment, especially in the presence of divalent ions like Mg ^2+^ and Ca ^2+^, could be orchestrated along the well-defined hydration shell of these ions. This suggests that the spatial arrangement and interactions of ThT molecules, influenced by the surrounding ionic environment, play a pivotal role in determining their lasing behavior.

The pronounced peak intensity of the complex in solutions enriched with magnesium and calcium provides further empirical support (lower panels in the inset images in Figure 4c,d. It underscores the role of divalent ions in amplifying the organization of ThT molecules within the hydration shell. This enhanced alignment of the orientation of the dipole moments of the dye is conducive to radiative decay, thereby optimizing the lasing output signal.

ThT photophysics was studied for G4-ThT in the presence of four cations. The SSF and TRF spectra show that cations play a key role in G4-ThT interaction by mediating structural dynamics and the stabilization of G4 (Figure S2 and Table S1). dsDNA can exist independently of salts. However, the formation and stability of G4 structures are intrinsically dictated by the presence of specific cations. These ions settle within the central channel formed by the stacked guanine tetrads of G4, providing both electrostatic stabilization and facilitation of the unique topology of these quadruplexes.

Mg ^2+^ stabilizes the G4 structure because it interacts strongly with the electronegatively charged pockets within the G4 structure, reinforcing its stability and formation. This unique role of magnesium in G4 stabilization was further proved by experimental lasing results. Lasing is exclusively observed in the presence of Mg ^2+^, while other salts fail to produce a similar effect. Thus, Mg has a unique role in shaping the G4 structure by enhancing the stability and inducing lasing.

**Figure 5:**
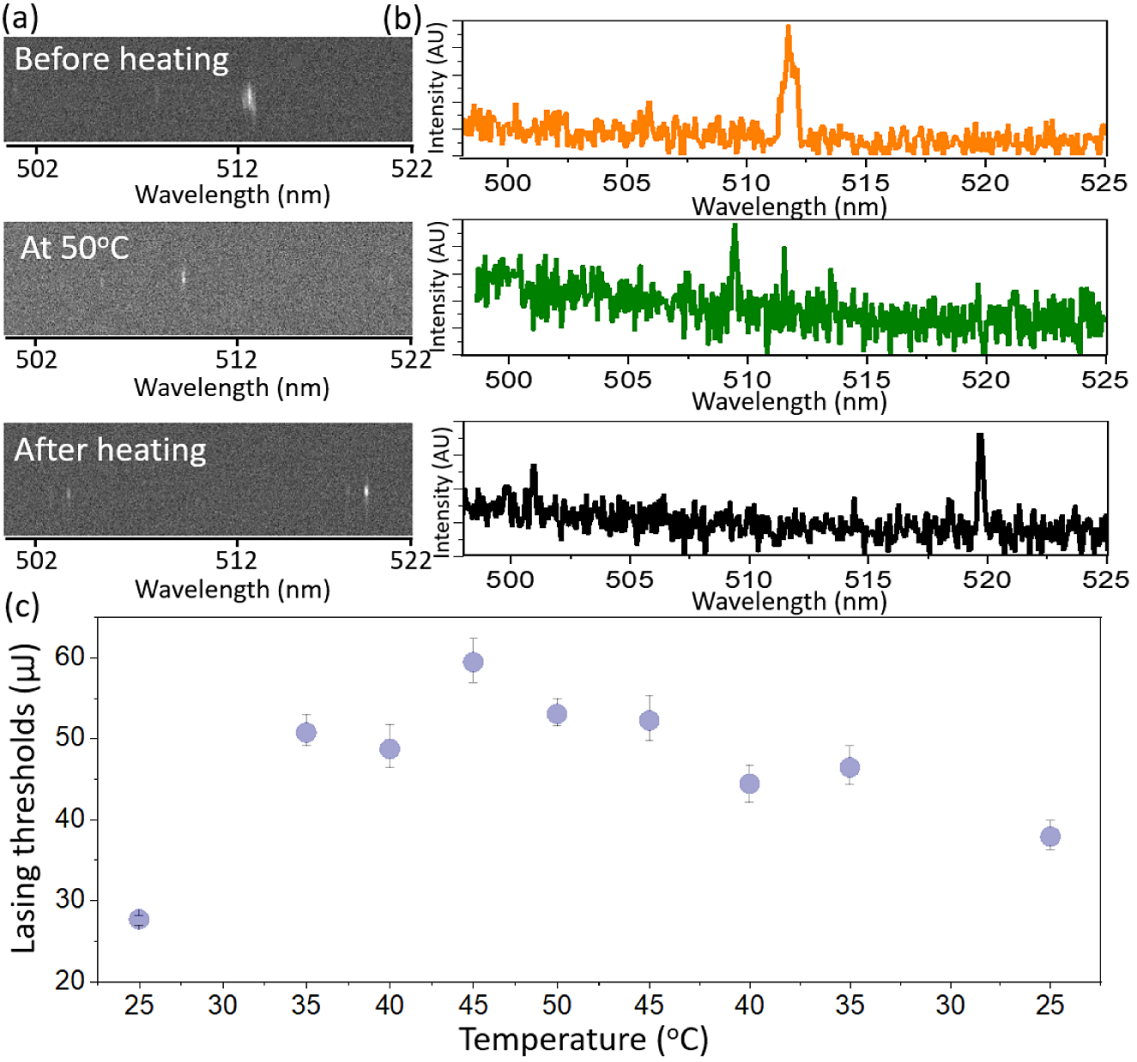
Visualization of lasing in ThT-stained G4 by Mg^2+^ stabilization: (a) top-view lasing images captured at different thermal stages, (b) spectral shifts in lasing signals during thermal processing, and (c) lasing thresholds observed in G4–ThT during melting experiments at specific temperature. *C*_ThT_ = 26.1 mM and *C*_G4_ = 44.8 mM. Excitation at 430 nm.

Lasing in the G4-ThT complex containing Mg^2+^ can be explained by the specific conformation that G4 adopt in the presence of Mg^2+^. The propeller-type structure has guanine bases oriented to resemble the propeller blades.^46^ This unique conformation might offer specific optical properties that are conducive for lasing. The absence of lasing in complexes containing K^+^, Na^+^, and Ca^2+^ suggests that G4 is not dependent on the presence of ThT in hydration shells. The lasing effect of G4 has a nuanced performance. The propeller-type G4 structure induced by Mg^2+^ initiates specific ThT ring alignment relative to each other for interaction and induces lasing. Thus, it is plausible that the chemical configuration of the dye stacked in guanine tetrads is more responsible than macroviscosity or microviscosity effects. The adoption of a specific chemical configuration in the presence of Mg^2+^ facilitates the necessary conditions for population inversion and subsequent lasing. This explanation is supported by the G4 partial melting experiment, where G4-ThT was heated stepwise to 50 °C followed by slow annealing (Figure 5).

G4 has a native Mg-stabilized propeller-type structure before heating. ThT molecules are probably aligned or stacked within the guanine tetrads in a specific manner to induce a lasing threshold at the 27.7 μJ pump fluence. Heating to 50 °C induces partial melting and destabilization of the G4 structure. This disruption alters the specific alignment of the aromatic rings of ThT in the guanine tetrads. Thus, the electronic interaction less favorable for lasing is altered, and the lasing threshold is increased significantly to ∼55 μJ. Lowering the temperature gradually refolds the G4 structure, which decreases the lasing threshold. However, the threshold after annealing at 20 °C is 38 μJ, indicating that the exact initial configuration is not regained during refolding. The G4 structure formed some kinetic traps or alternative folded states during regaining. Thus, the specific alignment of ThT rotor rings is not perfectly restored to its initial state, thereby reducing the lasing threshold (indicating a more favorable environment than the heated state) but remaining higher than that in the preheated sample.

The chemical configuration of ThT in Mg-stabilized G4 suggests that the specific alignment or interaction of ThT within G4 is sensitive to thermal perturbations. As the lasing threshold does not return to its original value on cooling, it implies that the initial and most favorable configuration of ThT within G4 is delicate and might not be easily regained after disruption.

In conclusion, while other cations can stabilize the G4 structure, the specific propeller-type conformation induced by magnesium seems to be the most favorable for lasing in G4-ThT system. This highlights the intricate interplay between the molecular structure and cation-induced conformational changes that are expressed in the ThT optical outputs. The G4 partial melting experiment provides convincing evidence that the lasing behavior of ThT depends on the specific chemical configuration within the Mg-stabilized G4 structure. Furthermore, a unique spatial arrangement of ThT molecules in the propeller-type G4 might lead to optimal overlap of their electronic transition dipoles, promoting efficient radiative decay and enhancing the lasing efficiency.

## Conclusions

The multifaceted interplay among alkali cations Na^+^, K^+^, Ca^2+^, and Mg^2+^ and their modulatory effects on ThT interaction within DNA was investigated. The findings underscored the distinct emission characteristics of ThT, which were contingent upon the presence and type of specific cations when interacting with dsDNA or G4. A distinctive emission behavior of dsDNA–ThT could be attributed to (i) a confluence of direct binding interaction between ThT and dsDNA and (ii) steric hindrance phenomena occurring within the DNA hydration shell. Steric hindrance was influenced by the surrounding alkali cationic environment, which was particularly pronounced in the presence of divalent cations where LD showed no binding to DNA but SSF was detected. A lasing effect was observed in DNA–Mg^2+^ and DNA–Ca^2+^ systems with a notably higher intensity in the presence of Ca^2+^ and Mg^2+^ than in the presence of K^+^ and Na^+^, suggesting a more organized arrangement of ThT molecules within the Ca^2+^- and Mg^2+^-mediated hydration shells. The organization of dye molecules and their respective dipole moments induced population inversion.

Furthermore, the G4 structure presented a unique scenario where lasing was exclusively observed in the presence of Mg^2+^. This observation accentuated the critical role of the parallel propeller configuration of G4 in fostering optimal conditions for ThT-mediated light amplification.

Overall, the results provided a deep understanding of the complex interplay among ThT, DNA, and various cations in dilute and condensed phases. The distinct behavior observed across different cations and DNA concentrations highlights the importance of the local microenvironment, specific ion effects, and chemical configuration of ThT rings in modulating the ThT optical readouts. The insight highlights the indispensable role of ThT as a cornerstone probe in advanced biophysical and molecular research endeavors.

## Supporting Information Available

Schematic representation of the experimental configuration employed for investigating the Fabry–Perot cavity lasing, fluorescence spectra and decays of ThT in the presence of calf thymus DNA and G4 and different cations, and table of lifetimes of ThT stained G-quadruplex (PDF)
