# Supplementary material for: Role of Alkali Cations in DNA–Thioflavin T Interaction
Source: J Phys Chem B. 2024 Jun 4;128(31):7520–9. doi: 10.1021/acs.jpcb.4c02417 (PMC11317975; doi:10.1021/acs.jpcb.4c02417)
Supplement: Supplementary file 1 — jp4c02417_si_001.pdf [file jp4c02417_si_001.pdf]

Role of Alkali Cations in DNA–Thioflavin T Interaction

P. Hanczyc*

Institute of Experimental Physics, Faculty of Physics, University of Warsaw, Pasteura 5, 02-093 Warsaw, Poland

Correspondence to: *piotr.hanczyc@fuw.edu.pl

Additional Figures

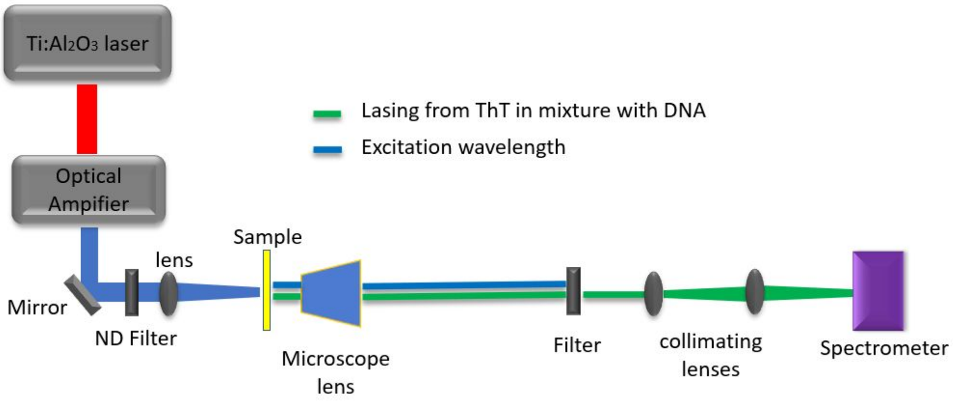

Fig. S1 A schematic representation of the experimental configuration employed for investigating the Fabry-Perot cavity lasing.

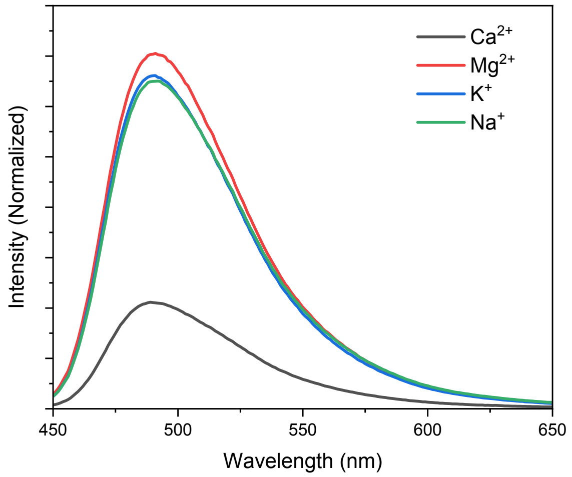

Fig. S2 Fluorescence spectra of ThT in presence of G4 and different cations Ca^{2+} (black), Mg^{2+} (red) K^{+} (blue), Na^{+} (green).

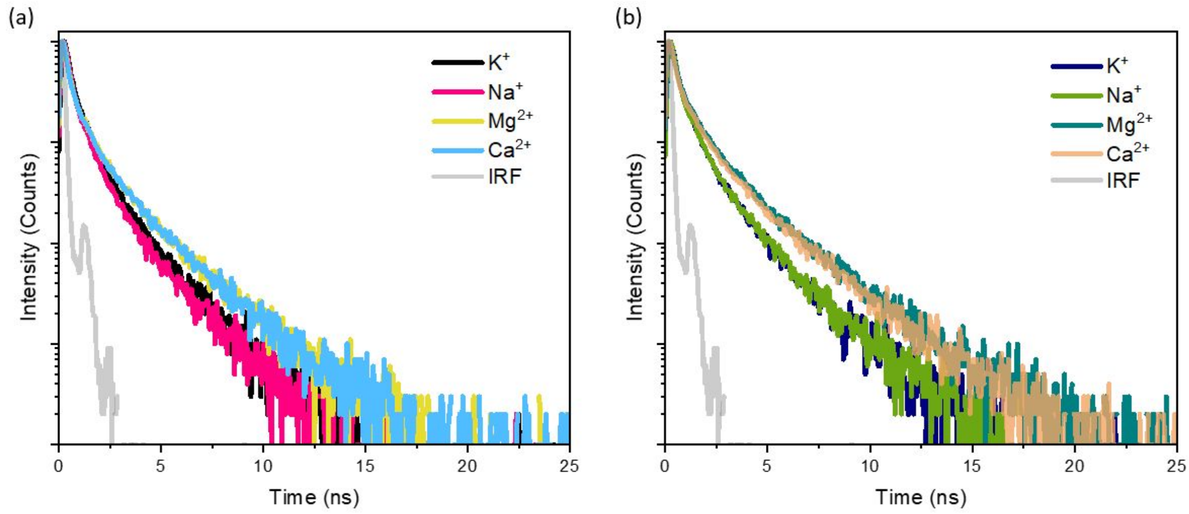

Fig. S3 Fluorescence decays ofcalf thymus DNA stained with ThT in presence of alkali cations. (a) C_{DNA} = 0.78 mM and (b) C_{DNA} = 1.56 mM C_{ThT} =13 pM , concentration of cations was 20 mM.

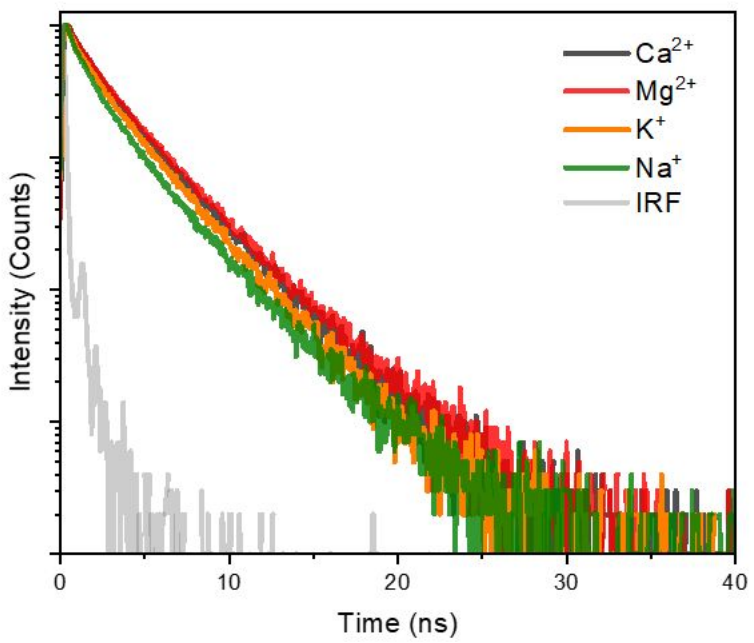

Fig. S4 Fluorescence decays of G-4 DNA stained with ThT in presence of alkali cations. C_{DNA} = 0.78 mM C_{ThT} =13 pM, concentration of cations was 20 mM.

Tab. S1 Lifetimes of ThT stained G-quadruplex with 20mM salts. excitation 430 nm, emission was collected at 490 nm

	Lifetime		
	τ_1 (ns)	τ_2 (ns)	τ_{avg} (ns)
Na^+	1.03 ±0.01 (0.37)	3.01 ±0.01 (0.63)	2.28
K^+	0.94 ±0.01 (0.23)	2.99 ±0.01 (0.77)	2.51
Ca^{2+}	1.33 ±0.01 (0.33)	3.33 ±0.01 (0.67)	2.67
Mg^{2+}	1.11 ±0.01 (0.55)	3.30 ±0.01 (0.45)	2.78
